# Patient perspectives on health care provider practices leading to an axial spondyloarthritis diagnosis: an exploratory qualitative research study

**DOI:** 10.1186/s12875-021-01599-2

**Published:** 2021-12-20

**Authors:** Kate L. Lapane, Catherine Dubé, Katarina Ferrucci, Sara Khan, Kristine A. Kuhn, Esther Yi, Jonathan Kay, Shao-Hsien Liu

**Affiliations:** 1 Division of Epidemiology, Department of Population and Quantitative Health Sciences, University of Massachusetts Medical School, 368 Plantation Street, Worcester, MA 01655 USA; 2 Clinical and Population Health Research Program, Graduate School of Biomedical Sciences, University of Massachusetts Medical School, Worcester, MA USA; 3 Division of Rheumatology, Department of Medicine, University of Colorado School of Medicine, Denver, CO USA; 4 Novartis Pharmaceuticals Corporation, East Hanover, NJ USA; 5 Division of Rheumatology, Department of Medicine, University of Massachusetts Medical School, Worcester, MA USA; 6 Division of Rheumatology, Department of Medicine, UMass Memorial Medical Center, Worcester, MA USA

**Keywords:** Delayed diagnosis, Quality of life, Focus groups, Spondyloarthritis, Patient preference, Back pain, Mass screening, Qualitative research

## Abstract

**Background:**

The average time to a diagnosis for people with axial spondyloarthritis (axSpA) is 7-10 years. Delayed diagnosis may result in increased structural damage, worse physical function, and worse quality of life relative to patients with a timely axSpA diagnosis. Understanding patient experiences may provide insights for how to reduce diagnostic delays.

**Objective:**

To provide foundational knowledge about patient experiences with healthcare providers leading to an axSpA diagnosis.

**Methods:**

We conducted an exploratory qualitative research study with six focus groups interviews with participants recruited from three rheumatology clinics within the United States (MA (*n* = 3); CO (*n* = 2); PA (*n* = 1)) that included a total of 26 adults (10 females, 16 males) with rheumatologist confirmed diagnosis of axSpA in 2019. Focus groups were ~ 2 h, audio recorded, transcribed, and subject to dual coding. The codes reviewed were in relation to the patients’ diagnostic experiences.

**Results:**

Patients described frustrating and lengthy diagnostic journeys. They recognized that the causes of diagnostic delays in axSpA are multifactorial (e.g., no definitive diagnostic test, disease characteristics, lack of primary care provider’s awareness about axSpA, trust). Patients described how doctors minimized or dismissed complaints about symptoms or told them that their issues were psychosomatic. Patients believed the healthcare system contributed to diagnostic delays (e.g., lack of time in clinical visits, difficulty accessing rheumatologists, health insurance challenges). Advice to physicians to reduce the diagnostic delay included allowing time for patients to give a complete picture of their illness experience, listening to, and believing patients, earlier referral to rheumatology, provision of HLA-B27 gene testing, and that physicians need to partner with their patients.

**Conclusions:**

Patients desire a definitive test that could be administered earlier in the course of axSpA. Until such a test is available, patients want clinicians who listen to, believe, and partner with them, and who will follow them until a diagnosis is reached. Educating primary care clinicians about guidelines and referral for diagnosis of axSpA could reduce diagnostic delay.

## Background

Axial spondyloarthritis (axSpA), including ankylosing spondylitis and non-radiographic axSpA, is characterized by waxing and waning symptoms of chronic inflammatory back pain with sacroiliac joint involvement [1]. Patients with axSpA often experience low back pain as their initial symptom [2]. Estimates of the prevalence of axSpA have been researched and are heterogeneous across place and time [1, 3]. In the United States, population-based estimates of the prevalence of axSpA are ~ 1% [4, 5]. This can cause further challenges in a primary care setting where back pain is a common presenting symptom [6], but patients with axSpA appear infrequently. Thus, differentiating common mechanical low back pain from inflammatory back pain can be complicatedfor primary care clinicians [7].

.Referred to as the ‘lost tribe’ [8], people with undiagnosed and untreated persistent inflammatory back pain suffer throughout their unacceptably long journey to a diagnosis (average time to diagnosis 7 to 10 years) [9–13]. Patients with axSpA often experience depression and desperation associated with their prolonged search for diagnosis and treatment [14]. The delay in diagnosing axSpA contributes to the increased economic burden, both for patients [15] and for the healthcare system [16]. A recent systematic review revealed that axSpA patients for whom the diagnosis was delayed had more structural damage, worse physical function, and worse quality of life than those with a timely diagnosis [17]. Early diagnosis of axSpA is critical, since early initiation of treatment lowers the likelihood of disease progression [18, 19] and reduces the extent of disability [20].

.Inadequate awareness and misconceptions about axSpA among primary care clinicians contributes to its delayed diagnosis, as do lack of diagnostic criteria, misinterpreted biomarkers, and difficulties with ordering correct imaging [8]. Primary care providers have agreed that improvements in screening for axSpA are needed and that there may be a role for a screening tool in the primary care setting [7]. Obtaining insights from axSpA patients about their diagnostic experiences is useful to inform the development of strategies to reduce delay in diagnosing axSpA. For this study, we sought to provide foundational knowledge about patient experiences during the journey to being diagnosed with axSpA. Also, we sought to understand patient perceptions regarding successful and effective approaches to screening for axSpA.

## Methods

The University of Massachusetts Medical School (UMMS) Institutional Review Board approved this study and granted a HIPAA waiver. A reliance agreement was provided by the University of Pennsylvania. All participants provided informed consent.

### Study design

**Fig. 1:**
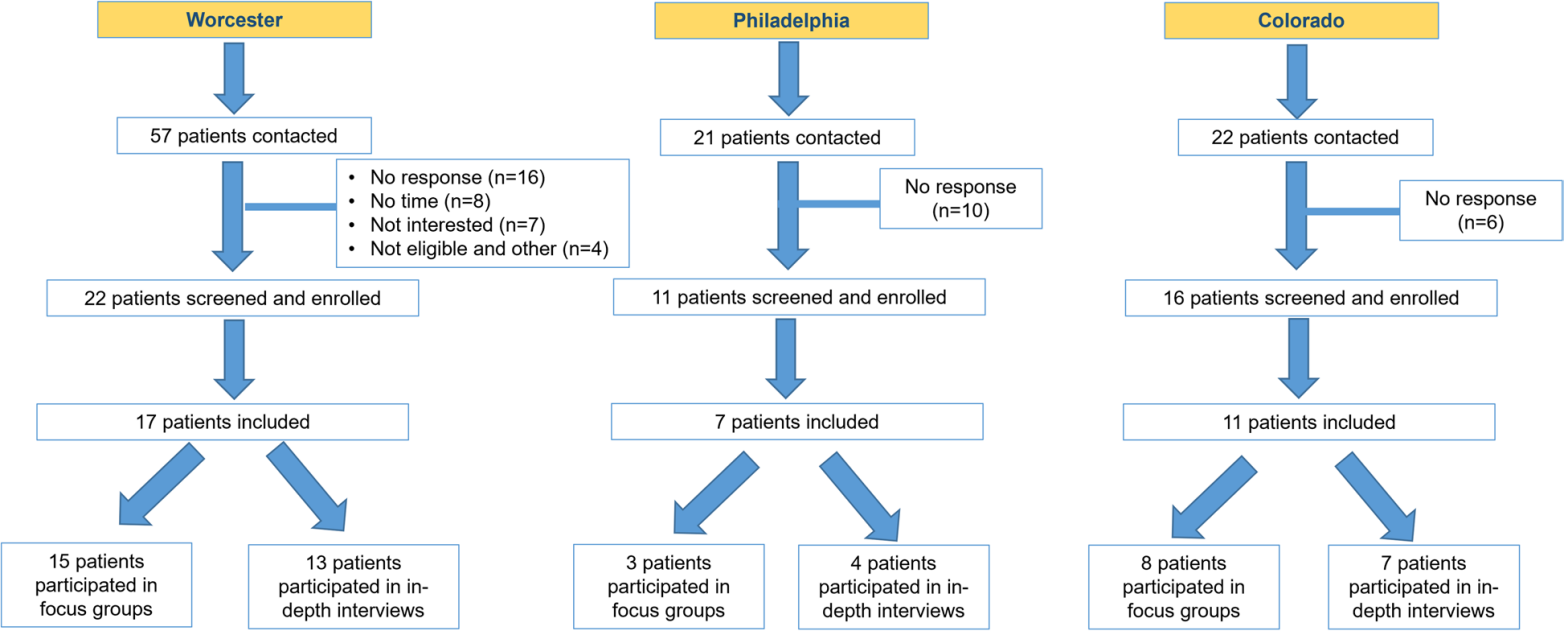
Patient Recruitment Flow Chart

We performed an exploratory qualitative research study in accordance with best practices [21], in which we conducted six focus groups. Our target population is US adults who have a rheumatologist confirmed diagnosis of axSpA. Also, we utilized purposeful sampling to target a variety of times from when patients first told their physician about symptoms to diagnosis ranging from early (< 2 years) mid (3-7 years) and late (> 7 years). We collaborated with three rheumatologists practicing in Worcester MA, Philadelphia PA, and Aurora CO. Locations were selected to provide patients from different regions in the United States where Rheumatologists were contracted. Rheumatologists followed our recruitment guidelines by alerting potentially qualifying patients. After an opt out period, the rheumatologists provided contact information to UMMS for those patients who were interested in participating. Eligible patients aged ≥18 years had a clinical diagnosis of axSpA verified by the referring rheumatologist and were able to provide informed consent (verified by the referring rheumatologist). People were excluded if they were unable to participate in a two-hour focus group, unwilling to be audio recorded, or did not speak English. One individual was no longer interested and withdrew from the study. Figure 1 depicts how patients were recruited to the study to participate in either an in-depth interview or focus group. A total of 57 patients from Worcester, MA, were contacted. Of those, 22 patients were screened and enrolled. Seventeen patients participated in the study (either focus group or in-depth interview) and 15 patients participated in the Worcester, MA focus group sessions. Twenty-one patients from Philadelphia, PA were contacted and 11 patients were screened and enrolled. Seven patients were included in the study and 3 participated in the focus group session. Twenty-two patients from Aurora, CO were contacted to participate in our study and 16 patients were screened and enrolled Of those 11 patients were included in the study and 8 patients participated in focus groups. The UMMS team confirmed subject eligibility and scheduled participants to participate in one of six focus groups from March 2019 to June 2019: three in in Worcester, MA (March 2019), two in Aurora, CO (April 2019), and one in Philadelphia, PA (June 2019). In Worcester, MA, the first focus group contained 7 participants. The second and third focus group sessions contained 4 participants each. In Aurora, CO, the first focus group interviewed 5 participants and the second focus group contained 3 participants. In Philadelphia, PA, 3 participants participated in the focus group.

### Conduct of the focus groups

A multidisciplinary team developed the focus group guides based on a scoping literature review [22–30], input from members of the sponsor’s research and two rounds of clinical review by the collaborating rheumatologists [31]. Focus group topics included: early symptoms, diagnostic journey, and advice for primary care doctors on how to reduce axSpA diagnostic delay (Table 5 in Appendix 1). CD, AB and KF had contact with participants during recruitment. Two members of the UMMS research team were present at each focus group, with one author (CD - Associate Professor (female)) who teaches graduate-level qualitative method courses moderating the session. A research assistant set up and tested dual audio recording devices, placed table signs that listed the main topics, handled collection of forms, surveys, and distributed $100 cash cards as (compensation for time and travel). A light meal was provided. At the beginning of the focus group, CD introduced herself and her role. A fact sheet detailing the rights and responsibilities of research participants was reviewed before beginning the discussion. The guideline incorporated semi-structured and open-ended questions to elicit relevant information. At the close of the focus group, participants completed a short background survey to determine if they wanted the opportunity to provide feedback on the draft report and/or would like to receive copies of manuscripts emanating from this research.

### Data processing

Audio recordings were uploaded to a secure UMMS server. CD selected the recording with the best audio quality and uploaded it via secure ShareFile to a HIPAA compliant transcription service. Verbatim transcripts were reviewed for accuracy, errors were corrected, names were replaced with generic references (“my doctor” instead of “Dr. X”), un-identified speakers were labeled, and (if possible) indistinct comments were clarified. Research assistants replaced participant names with ID numbers and then imported the transcripts into NVivo 12 [32]. (QSR International https://www.qsrinternational.com/nvivo/nvivo-products). One author (CD) developed a coding structure based on the focus group topics and her initial impressions of focus group content.

### Analysis

We conducted a thematic analysis of qualitative data [33]. A thematic analysis was conducted using a coding start-list which was derived from the focus group protocol [33]. New codes were also added as they emerged during coding. Overall, an iterative thematic analysis was applied [33]. First, research assistants read all focus group transcripts, listened to all focus group recordings at least once, and wrote a summary. Two members of the research team independently then coded each focus group transcript into one NVivo 12 project. Code reports were generated for each thematic code. Two team members independently prepared summaries of impressions for each code, tagging quotations reflective of the summaries. Impressions were discussed for each code and sub-code and consensus compilations were finally crafted to consolidate the duplicate code summaries and original code reports. Compilations and summaries served as source documents. This paper focuses on those codes relevant to the diagnostic experience. A two-page preliminary report of our findings was emailed on August 7, 2019, to those participants who had requested it.

## Results

### Sample characteristics

The six focus groups included 26 patients with axSpA (range: *n* = 3-7 participants; average length 108 min (range: 80 to 128 min). Most participants were men (*n* = 16) (Table 1). The average age of participants was 53.5 years (range: 21 to 76 years old), the average age at first symptom was 22.8 years (range: 10 to 55 years old) the average age at which the patient first told their primary care physician about symptoms was 24.4 years (range: 10 to 55 years old) and the average age at diagnosis was 34.8 years (range: 15 to 65 years old) Most participants were non-Hispanic White and 62% had a college degree or higher. Almost all (> 90%) wanted the opportunity to give feedback on preliminary findings or publications emanating from the research.

**Table 1:** Characteristics of patients with axial spondyloarthritis participating in focus groups

	Overall (*n* = 26)
	*mean (standard deviation)*
Age in years	53.5 (15.1)
Age at first symptom	22.8 (10.1)
Age first told doctor	24.4 (10.7)
Age at diagnosis	34.8 (12.7)
Time from first symptom to first tell doctor	1.7 (2.7)
Time from first told doctor to diagnosis	10.4 (9.1)
Time from first symptom to diagnosis	12.0 (9.5)
	*percentage*
Women	38.5
Race/ethnicity	
White	76.9
Black	7.7
Hispanic	3.9
Other	11.5
Education	
High School or less	0
Some college	38.5
College graduate	30.8
Graduate degree	30.8
Medications	
NSAIDS	46.2
Corticosteroids	0
TNF-alpha inhibitors	42.3
IL-17 inhibitors	3.9
IL 12/23 Inhibitors	19.2
Opioids	7.7
DMARDS	0

Percentages may exceed 100% due to rounding

### Findings of common themes

Common themes included the lengthy trial and error approaches that led to misdiagnosis and initiation of failed therapies; dismissal of intermittent symptoms by healthcare providers; and the need for patients to research their own diagnosis because providers stopped trying to make an accurate diagnosis (Table 2). Although some participants experienced a timely diagnosis, many others described long circuitous journeys to search for a diagnosis, which involved seeing primary care and specialist physicians, chiropractors, and physical therapists. Patients estimated time from first symptoms to being diagnosed averaging 12 years (range 0-37 years) and time from their first mentioning symptoms to a physician to being diagnosed averaging 10 years (range 0-30 years). Some participants had been prescribed therapeutic trials of many different medications for symptom relief, in the absence of a clear diagnosis.

**Table 2:** Shortcomings of existing screening procedures

Theme	Representative quotes
Lengthy trial & error approaches	*“I went on every different drug you could think of, you know, just – you got this. And then every year they’d give me a new disease. Oh, you got this disease, sacral plexus, you know, they’d tell me whatever and it was like, you can’t even spell it, some of the diseases they’d give me. And every year I’d have a new disease…” (46 year old man)*
Intermittent symptoms need to be taken into consideration	*“It would be a problem for -- a severe problem for, like, 2 to 4 days, a little bit of an issue for anywhere from 2 to 3 weeks. I’ve learned these things pretty much last 2 to 3 weeks, and then they go away if they’re going to go away.” (47 year old man)*
Symptoms minimized or disregarded	*“Like, I would get sent to one doctor, and I had x-rays taken, and, oh, well, there’s nothing wrong with you. I had one doctor that told me it was all in my head… I was very, very upset when she told me, everything, all my pain, everything was in my head, I was crazy.” (48 year old female)*
Early symptoms can be due to many other things	*“it wasn’t the first thing that anyone thought, you know, because it could be ten other things.” (34 year old male)*
Doctors give up when they can’t figure it out	*“So, I went to the emergency room and they were going to send me home again that Friday night because they said we can’t really figure out, like, nothing seems to be wrong with you” … “It’s way more disheartening, frustrating, and discouraging to be told constantly there’s nothing wrong with you.” (34 year old male)*
Patients are having to do the legwork	*“So I had done a lot of research on different things and I just said to my doctor do you think because I have autoimmune any of that is ankylosing spondylitis. And she said huh, she said, well, you know it’s usually males in their late 20s, 30s, and she said… But she said let’s test you… I tested positive for the HLA-B27, then she sent me to the rheumatologist who then did the x-ray and saw the sacroiliitis and put it together.” (61 year old female)*

Participants appreciated that their early symptoms could have been attributed to a wide variety of other conditions, making it difficult for physicians to diagnose them. Yet, participants wanted physicians to reach a diagnosis as rapidly as possible so that they could begin appropriate treatment. The slow, progressive nature of axSpA, with symptoms that could not readily be attributed to an antecedent event, compelled patients to impress upon their physicians that something was genuinely wrong. To emphasize the seriousness of their illness experience, patients often needed to use dramatic language to convey the urgency and legitimacy of their symptoms (e.g., pain level of 10 as “suicidal” (46 year old male); assigning a 1-10 pain scale rating for a muscle spasm “25… it’s horrible” (54 year old male)).

The waxing and waning patterns of pain resulted in some patients delaying seeking medical care and confused some physicians. Participants felt strongly that primary care physicians should not stop trying to establish a diagnosis, regardless of how challenging it might be. In fact, when primary care physicians abandoned their diagnostic quest, patients experienced this as being profoundly negative. During the typically lengthy time that participants waited to be diagnosed, participants experienced frustration and mental suffering. Many participants left with several of their questions not being answered and/or not being believed or taken seriously. Participants were eventually diagnosed with axSpA, but for many of them, this was the result of their tenacity and/or that of their family members to determine the source of their suffering. Some participants conducted their own research and were confident enough to challenge their physicians. However, some resented having had to resort to researching their symptoms on their own.

### Findings from patient perspectives on improving the screening process

Participants discussed their early experiences from when their symptoms emerged, and they started seeking explanations and relief from their primary care physicians (Table 3). A recurring theme was that participants felt that they were neither “heard” nor believed. Participants described a variety of negative experiences ranging from primary care physicians minimizing or dismissing their complaints to primary care physicians concluding that their symptoms were imaginary. Participants’ advice to primary care physicians was straight-forward: allow patients to explain their symptoms, listen to them, and believe them. Further, participants highly valued clinicians who were there for them and persistent, who partnered with them in the diagnostic quest, and who followed through to make a correct diagnosis. Beyond listening to and believing the patient, some participants stressed the importance of primary care physicians gaining a complete picture of the patient’s experience with their illness.

**Table 3:** Patient perspectives on improving the screening process for axial spondyloarthritis

Theme	Representative quotes
Listen and believe the patient	*“But there’s like that famous joke that -- the tombstone that says I told you I was sick. I mean, that’s the way I felt was like I told you something, like, it wasn’t just complaining” (34 year old male)*
Do not come to premature closure when the patient does not fit the typical profile	*“Be willing to listen to your patient, do the diagnostics, do the research. If what you’re doing isn’t working, you know, don’t follow that same path that’s not working. Take a step back. Talk to your peers. I mean, we say it at work, phone a friend. There’s got to be other people you can talk to if you’re not helping your patient. Refer them out. Don’t make your patient suffer for something that you’re not sure about.” (41 year old female)*
Find a more definitive test /use HLA-B27	*“maybe it points out that there should be more research? … And finding out a marker that can be identified and then they can treat it, but if you just sort of well, it could be this, it could be that -- something definitive that would help in a diagnosis.” (75 year old male)* *“I went to see my primary care and I told my primary care [I was] having a sacroiliac pain with uveitis, I really want you to check my HLA-B27. So he ordered it, it came back positive, and then sent me to the rheumatologist and that’s how I got the diagnosis, basically 25 years later.” (45 year old male)*

Participants articulated the desire for a definitive diagnostic test. Most reported that they had been tested for the HLA-B27 antigen and were positive. Participants would have preferred to have received HLA-B27 testing earlier in the diagnostic process. Although many understood that this test did not establish a diagnosis of axSpA definitively, it often confirmed their final diagnosis.

### Findings regarding barriers to implement improved screening and early detection

Participants suggested that primary care physicians would benefit from additional education about axSpA (Table 4). Many participants reported that their primary care physicians admitted to them that they did not know what might be causing their symptoms. Conversely, some participants described primary care physicians who kept researching their symptoms and educating themselves to figure out what was happening to their patient. Some participants stressed the importance of a partnership between the provider and the patient but acknowledged that this was difficult to achieve during appointments of short duration. Some participants spoke of physicians who assumed that they were exhibiting drug seeking behavior, hypochondriasis, or psychosomatic illness. Since axSpA patients are often young and otherwise healthy, physical findings might not have been present at the time of their clinical encounter.

**Table 4:** Barriers that need to be addressed to implement improved screening and early detection

Theme	Representative quotes
Additional education for primary care doctors	*“The education of the physicians. The ankylosing spondylitis is one of the most common forms of arthritis but yet they don’t look at it as -- and that is something that they need to be educated on.” (65 year old female)*
Improve physician empathy, persistence, relationship with patient	*“The doctor/patient relationship is huge.” (54 year old male)* *“if you have a good relationship with the doctor and you’ve done your homework It should be a partnership, and that’s what I would recommend” (66 year old male)*
Physician assumptions: drug seeking, hypochondriasis	*“Yeah, the pain, and they look at you… what do you mean pain, they just look like they have no idea that you’re in pain… And they look at you like well, nowadays, it’s even worse with the opioid crisis…” (65 year old female)*
Improve access to specialists and care coordination	*“I tried to make a point to emphasize the care coordination call because that, when all these doctors talked to each other, that’s when like everything kind of clicked… I think more care coordination or just -- or just maybe something where doctors talk to people who are not in their field, you know?” (34 year old male)*
Not enough rheumatologists	*“There’s limited rheumatologists and it’s hard to get in with them unless they know something’s wrong with you” (28 year old female)*
Make time and information available	*“When I go to a doctor if I’ve got questions I write them down so I don’t forget, okay? And I had a doctor ready to leave the room after an exam and I said wait a minute, I’ve got more questions. And he said I’ve got more patients…” (73 year old male)*

Many participants lamented the long wait time to see a specialist. Patients thought that referral to a specialist could accelerate the time to diagnosis but perceived that the shortage of rheumatologists contributed to these delays. Patients often described their visits with a rheumatologist, once they finally took place, as having been short and hurried. However, in at least one case, coordination of care among treating physicians, including a rheumatologist, resulted in effective diagnosis and treatment.

Overall, participants conveyed the feeling that they had been widely misunderstood by many physicians, which for many resulted in a lengthy delay in being diagnosed and receiving effective treatment. Nearly all participants described experiences in which they had to advocate for themselves and/or rely on the advocacy of family members to accelerate the diagnostic process. This often took the form of “chasing a diagnosis” over the course of years. Some felt that they had to diagnose themselves, receiving confirmation only when a doctor agreed to proceed with HLA-B27 testing or referral to a rheumatologist.

*“Doctors, I mean, they care, but they don't. Like, if you don't just, you know, get to the point, be there for yourself, like, advocate for yourself, they're just going to then walk out the door and say nothing's wrong.” 28 year old female*

## Discussion

Through a rigorous analysis of six focus groups, conducted in three different geographic locations, we found that patients with a confirmed diagnosis of axSpA described lengthy and frustrating journeys toward a diagnosis. Participants estimated time from first symptoms to being diagnosed averaging 12 years (range:0 years to 37 years) with time from their first mentioning symptoms to a physician to being diagnosed averaging 10 years (range 0-30 years. Meaning participants could potentially wait an average of 2 years to describe symptoms to their physician. Patients recognized that the causes of diagnostic delays in axSpA are multifactorial and include absence of a definitive diagnostic test, indistinct disease characteristics (e.g., intermittent, vague symptoms), and factors related to providers (e.g., lack of awareness about axSpA, time, trust), and the healthcare system (e.g., brief duration of clinical visits, limited access to rheumatologists). Patients described primary care physicians who minimized complaints about symptoms that were sometimes severe, who dismissed their complaints completely, or who told them that their issues were psychosomatic. Patients with axSpA articulated clear advice to physicians: provide patients with adequate time for them to give a complete picture of their experience with their illness, listen to them, believe them, and partner with them throughout the sometimes-lengthy diagnostic process.

Many participants in our study commented on their primary care provider’s lack of awareness about axSpA, which is consistent with previous research [20]. Patients with axSpA advocated for the development of a definitive test that could be administered earlier in the course of the disease. Many advocated for wider use of HLA-B27 testing earlier in the diagnostic journey because positive findings led to their eventual diagnosis. Participants exhibited variable understanding of the role of HLA-B27 testing in the evaluation of patients with axSpA: some believed the test to be diagnostic of axSpA, and others recognized that patients may not be diagnosed when they should in the absence of the HLA-B27 marker. Structural changes of axSpA evident on plain radiographs may take years after the onset of initial symptoms to develop [34, 35]. Magnetic resonance imaging is available to assess for osteitis; yet, the delay in diagnosis of non-radiographic axSpA is also unacceptably long [35]. Diagnostic guidelines have improved detection of some [36], but not all diseases [37, 38]. Patients articulated that the development of diagnostic guidelines (as there are only axSpA classification criteria) for axSpA and early referral to a rheumatologist could reduce diagnostic delay. The extent to which axSpA diagnostic guidelines would reduce the time to axSpA diagnosis remains to be seen.

System-level factors were perceived by patients with axSpA as contributing to diagnostic delay. Consistent with previous research [7, 39, 40], patients in our study noted that the brief duration of primary care clinical encounters contributed to diagnostic delay. In a health maintenance organization, the average length of a primary care visit was 27 min [41]. In primary care settings, only 5 min is spent discussing the primary complaint [42]. Patients in our study felt that had primary care physicians been given enough time to hear their entire illness experience, the duration of their diagnostic delay would have been reduced. Since patients with axSpA often have multiple co-morbidities [43], the time allotted for a primary care visit may not be long enough to address their axSpA symptoms. However, increasing the length of primary care visits is problematic since it is impacted by the number and medical complexity of the patients in the provider’s panel [44], and the requirement to use an electronic health record [41]. In our study, patients who researched their own symptoms and advocated for themselves were able to arrive at a diagnosis of axSpA despite the relatively brief duration of primary care encounters. Patients in the current study acknowledged that limited access to rheumatologists contributes to the delay in diagnosis of axSpA. The average wait for a rheumatology appointment is 4 months [45]. Nevertheless, despite the increasing demand for the services of rheumatologists, the number of practicing rheumatologists has been projected to decline [46–48]. These projected shortages will likely increase the average wait time and/or result in excessive patient travel time to consult with a rheumatologist [49].

.Most axSpA patients are eventually diagnosed by non-rheumatologists [8, 50]. Patients with axSpA often fit the profile of “difficult” patients as they often present with a “chronic course of multiple vague or exaggerated symptoms” [51]. “Difficult” patients are considered to be those with multiple symptoms, who are under stress [52], and may be angry, defensive or frightened” [51]. Such impressions may evoke negative feelings and aversive reactions among physicians, often resulting in compromised medical care [53]. However, patients in our study were satisfied with their primary care, even with protracted diagnostic journeys, *if* their physicians believed them and did not give up before a diagnosis was reached. These participants found comfort with their trusted physician and described deep appreciation for their efforts. Patients with axSpA in our study echoed what all patients want from primary care: “a trusting, longitudinal relationship with a competent, caring primary care provider who is committed to their well-being” [54].

### Strengths and limitations

Qualitative research provides insights that cannot be obtained by typical survey research or analyses of claims or electronic health records. We designed our guidelines to be aligned with best practices and we recruited participants from three geographic locations. All participants had received a diagnosis of axSpA, but the time since diagnosis was varied and sometimes was quite long. Information regarding the diagnostic journey was based on patient recall and the extent to which these experiences are reflective of patients without a confirmed diagnosis of axSpA is unknown. Most of the participants were highly educated and all spoke English. The experiences herein may not reflect those of patients with limited English proficiency or with lower levels of educational attainment. Finally, patients agreeing to participate in a focus group may have more confidence and ability to speak up and express their feelings and opinions. Patients who are more insecure or reserved might decline participation in a focus group and thus their views would not be represented.

## Conclusions

We found that patients with axSpA experienced frustration with their lengthy diagnostic journeys. As a result, they have had to become their own advocates. Patients want a definitive test that could be administered earlier in their disease course. Until such a test is available, patients want clinicians to listen to them, believe their symptoms, partner with them, and persist until a diagnosis is reached. Providing non-rheumatologists with axSpA diagnostic guidelines and recommendations for referral may reduce the delay in diagnosing patients with axSpA. Patients need a primary care physician advocate who can partner with them on their journey towards a diagnosis. Future studies should examine the usage of screening tools to identify axSpA patients in primary care centers.

## Data Availability

The data analyzed during the current study are not publicly available due to the nature of the qualitative data and the conditions of the informed consent received by our study participants. As such, we are unable to share the qualitative data from this study.

## Appendix 1

**Table 5:** Focus group questions

Focus Group Questions	
1. What do you call your arthritis and why?	
2. How did spondylitis start for you? What were your earliest symptoms?	
3. Before you were diagnosed, what kinds of things did you say to your doctor about it?	
4. Sometimes people with spondylitis wait a long time before finally being diagnosed. Sometimes 7 or 10 years. What was your experience? What was the diagnostic journey like?	
5. Written exercise: What do these terms mean to you? (chronic, stiffness, flare-up, acute) -- Discussion: How about these other terms: Psoriasis; Iritis; NSAID, uveitis, etc.	
6. Who has experienced “stiffness”? How would you describe it to someone who has never had it?	
7. How do you talk about pain to your doctor? When a doctor asks you how long you have had pain – what does that mean to you?	
8. Written Exercise: Look at the handout called “Your Arthritis – Side A.” Use the figure her and mark the spot of the **first pain, aching or stiffness**. Anyone want to comment? Now side B. Mark your pain, aching or stiffness that **affected your life the most.** Comments?	
9. What advice do you have for primary care doctors, family doctors etc.? Rheumatologists?	
10. If you could go back in time, how would you discuss your symptoms to help reach a diagnosis more quickly? Would you have done anything differently?	

## Appendix 2

Table 6

**Table 6:** Extract from focus group codebook

Name	Description
Early symptoms	First symptoms, symptoms prior to dx, symptoms leading to doctor visit, childhood symptoms
Told physician	Things patients told their doctor about their symptoms, before diagnosis, for diagnosis, after diagnosis
Failed therapy	Therapies tried and failed prior to diagnosis -- may include pharmacotherapies, physical therapy, chiropractic, acupuncture, different mattress, psych therapies, etc. etc.
Wrong diagnosis	Misdiagnoses prior to eventual diagnosis of SpA (example - “they thought it was a back sprain” -- “they even thought I had cancer as a child” “they told me it was because one leg was shorter than the other” etc.
Diagnosis	Experience with diagnosis and delay, Getting the SpA diagnosis, hearing the SpA diagnosis for the first time
Helped diagnosis	Things they helped get a diagnosis – Firing docs and getting new ones, self-advocacy, having an advocate doc, friends/family who advocated, persistence, HLA-B27, etc.
HLA-B27	Discussion of genetic predisposition, family history, results of the HLA-B27 test.
Impede diagnosis	Things that interfered with dx – Gender, MD or family disbelief, “drug-seeking”, No HLA-B27
Health system	Comments about insurance coverage, co-pays, access to services, pharmaceutical industry, etc.
Done differently	Things the patient might have done differently in retrospect - things they wish they would have done to get their diagnosis sooner.
Advice for physicians	Advice for primary care docs, rheumatologists, other docs

## Abbreviations

axSpa: Axial spondyloarthritis
UMMS: The University of Massachusetts Medical School
